# Dietary factors and DNA methylation-based markers of ageing in 5310 middle-aged and older Australian adults

**DOI:** 10.1007/s11357-024-01341-7

**Published:** 2024-09-19

**Authors:** Lachlan Cribb, Allison M. Hodge, Melissa C. Southey, Graham G. Giles, Roger L. Milne, Pierre-Antoine Dugué

**Affiliations:** 1 Precision Medicine, School of Clinical Sciences at Monash Health, Monash University, Level 3, MIMR, 27-31, Wright St, Clayton, VIC 3168 Australia; 2 Cancer Epidemiology Division, Cancer Council Victoria, Melbourne, VIC Australia; 3 Centre for Epidemiology and Biostatistics, Melbourne School of Population and Global Health, The University of Melbourne, Parkville, VIC Australia; 4 Department of Clinical Pathology, The University of Melbourne, Parkville, VIC Australia

**Keywords:** Diet composition, Diet quality, Biological age, Epigenetic clocks, Epigenetic ageing

## Abstract

The role of nutrition in healthy ageing is acknowledged but details of optimal dietary composition are still uncertain. We aimed to investigate the cross-sectional associations between dietary exposures, including macronutrient composition, food groups, specific foods, and overall diet quality, with methylation-based markers of ageing. Blood DNA methylation data from 5310 participants (mean age 59 years) in the Melbourne Collaborative Cohort Study were used to calculate five methylation-based measures of ageing: *PCGrimAge*, *PCPhenoAge*, *DunedinPACE*, *ZhangAge*, *TelomereAge*. For a range of dietary exposures, we estimated (i) the ‘equal-mass substitution effect’, which quantifies the effect of adding the component of interest to the diet while keeping overall food mass constant, and (ii) the ‘total effect’, which quantifies the effect of adding the component of interest to the current diet. For ‘equal-mass substitution effects’, the strongest association for macronutrients was for fibre intake (e.g. *DunedinPACE*, per 12 g/day − 0.10 [standard deviations]; 95%CI − 0.15, − 0.05, *p* < 0.001). Associations were positive for protein (e.g. *PCGrimAge*, per 33 g/day 0.04; 95%CI 0.01–0.08, *p* = 0.005). For food groups, the evidence tended to be weak, though sugar-sweetened drinks showed positive associations, as did artificially-sweetened drinks (e.g. *DunedinPACE*, per 91 g/day 0.06, 95%CI 0.03–0.08, *p* < 0.001). ‘Total effect’ estimates were generally very similar. Scores reflecting overall diet quality suggested that healthier diets were associated with lower levels of ageing markers. High intakes of fibre and low intakes of protein and sweetened drinks, as well as overall healthy diets, showed the most consistent associations with lower methylation-based ageing in our study.

**Supplementary Information:**

The online version contains supplementary material available at 10.1007/s11357-024-01341-7.

## Introduction

The role of nutrition in healthy ageing is widely accepted, with evidence from animal and human studies indicating a crucial role for diet in the processes of ageing and longevity [1]. Yet, the contribution of specific dietary components and compositions to human ageing has not been comprehensively described. Recently developed signatures of biological ageing, that is, the rate of age-related decline in the integrity of cells and organs [2], provide a promising avenue for characterising the influence of diet on healthy human ageing.

Many proxy measures of biological age have been developed, the most promising of which are those based on DNA methylation. While early ‘epigenetic clocks’ such as Hannum’s [3] and Horvath’s [4] aimed to predict chronological age with high accuracy, several recently developed methylation-based ageing markers — notably *PhenoAge* [5], *GrimAge* [6], *ZhangAge* [7], Dunedin Pace of Ageing Methylation (*DunedinPACE*) [8], and epigenetically estimated telomere length (*DNAmTL or TelomereAge*) [9] — aim to predict biological age more directly by incorporating a greater array of physiological information. Higher methylation-based age estimates, for a given chronological age, indicate relatively rapid biological ageing, a phenotype that has been associated with increased risk of disease and mortality [2, 10, 11].

Prior evidence indicates that these methylation-based measures of biological ageing are sensitive to dietary influences. In a pilot randomised controlled trial, Fitzgerald et al. [12] found a reduction in estimated methylation age (~ 3 years, based on Horvath’s clock) in those assigned to a comprehensive lifestyle intervention, including dietary change and phytonutrient and probiotic supplementation over 8 weeks, compared with no intervention. Another trial of 219 post-menopausal women followed over 24 months found that receiving dietary advice to follow a plant-based diet, with a low glycaemic load, low in saturated and trans-fats and alcohol, and rich in antioxidants, was associated with reduced *GrimAge* at the end of the study [13]. Cross-sectional studies have identified modest correlations between Horvath’s and an enhanced version of Hannum’s epigenetic clocks with select food groups [14], as well as associations between *PhenoAge* and *GrimAge* and dietary biomarkers, food group intakes [5, 6], and overall dietary quality [15]. Kim et al. [16] found that increased methylation-based age partly mediated the relationship between dietary quality and all-cause mortality. While previous studies have investigated the relationship between dietary quality and dietary components with methylation-based markers of ageing, most were limited to relatively few dietary exposures (or only indices of dietary quality), focused on first-generation methylation-based measures of ageing, and had relatively small sample sizes. Consequently, we sought to estimate cross-sectional associations between dietary composition, including macronutrient composition, food groups, dietary quality, and energy intake, and recently developed methylation-based markers of biological ageing.

## Methods

### Study sample

This cross-sectional study uses baseline data from the Melbourne Collaborative Cohort Study (MCCS), a prospective cohort study which recruited 41,513 participants to investigate the roles of diet and lifestyle in causing cancer and other non-communicable diseases [17]. Participants were recruited from the electoral rolls (registration to vote is compulsory in Australia) as well as media and community advertisements. Southern European migrants (5430 Italians and 4526 Greeks) were oversampled to extend the range of lifestyle exposures within the cohort. Most (99%) participants were aged between 40 and 69 years at baseline (1990–1994). Blood samples were collected at baseline in 99% of participants. The DNA sources for the present study included dried blood blots, peripheral blood mononuclear cells, or buffy coats for 70%, 28%, and 2% of participants, respectively.

The present study sample comprised MCCS participants selected for inclusion in any of seven previously conducted nested case–control studies of DNA methylation (sample selection is displayed in eFigure 2) [17–20]. All participants were free of cancer (or free of urothelial cancer in the urothelial cancer study) at the time of baseline blood draw.

All participants provided written informed consent, and the study protocols were approved by the Cancer Council Victoria Human Research Ethics Committee.

### DNA methylation

Methods relating to DNA extraction, bisulfite conversion, and DNA methylation data processing have been described previously [21]. Some participants were included in more than one of the underlying case–control data sets. Thus, replicated methylation measurements were available for this subset of individuals (*N* = 526, providing 1086 total measurements). Participants from the nested breast cancer case–control study were excluded from this analysis as methylation was measured using a different protocol from the other studies.

### Health and demographic data

Participants completed detailed questionnaires on lifestyle, demographic, and medical history. Smoking was self-reported as smoking status (current, former, never) and smoking pack years were calculated. Alcohol intake (g/day) was calculated based on the number and volume of alcoholic drinks consumed over the prior week. Height and weight were measured by trained personnel. The decile score of the socioeconomic index for areas (SEIFA), a neighbourhood-level metric of socioeconomic status created by the Australian Bureau of Statistics [22], was used as an indicator of socioeconomic status.

### Dietary exposures

The food frequency questionnaire (FFQ) was developed specifically for the MCCS based on weighed food records and has been described in detail previously [23]. In short, the FFQ consisted of 121 food items with frequency responses ranging from ‘never or less than once a month’ to ‘6 or more times per day’. Dietary exposures of interest were macronutrients (including fibre) and subcomponents, food groups, indices of dietary quality, total energy, and coffee and tea intake. Macronutrients and subcomponents consisted of protein, carbohydrates (sugar, starch and dextrins), fibre, and fat (saturated fat, monounsaturated fat, and polyunsaturated fat). Foods were categorised into groups: breakfast cereal, sweet cereals and confectionary (cakes, sweet biscuits, chocolate), savoury cereals (pies, pizzas), starchy foods (rice, bread, pasta, potatoes), savoury dairy (yoghurt, cheese, cream), sweet dairy (ice cream, custard, sweet milk drinks), eggs, butter, margarine, fresh red meat, processed meat, chicken, seafood, vegetables, fruit, legumes, fruit juice, sugar sweetened drinks, artificially sweetened drinks, nuts, spreads and dips, olive oil, and vegetable oil. Details of food groups, including constituent foods, are given in eTable 1. Daily intakes in grammes were calculated by multiplying the number of daily serves of each food group by a sex-specific portion size (derived from weighed food records) [23]. Nutrient intakes were calculated by multiplying the intakes of each item by the nutrient composition. Nutrient-composition data were derived from the Australian 1995 database [24]. Total energy intake was calculated from all food and alcohol sources.

The Dietary Inflammatory Index (DII®) [25], Mediterranean Diet Score (MDS) [26], and Alternative Healthy Eating Index-2010 (AHEI-2010) [27] were calculated as described in previous MCCS publications and used as measures of overall diet quality. Cups per day of coffee and black tea were calculated from frequency responses ranging from ‘never’ to ‘6 or more cups per day’.

### Methylation-based ageing outcomes

Markers of methylation-based ageing (*PCGrimAge*, *PCPhenoAge*, *DunedinPACE*, *ZhangAge*, *TelomereAge*) were calculated using the R package dnaMethyAge [28]. *PCGrimAge*, *PCPhenoAge*, and *DunedinPACE* are recent iterations of *GrimAge*, *PhenoAge*, and *DunedinPoAm* [29], respectively, which have improved test–retest reliability compared to their predecessors [30, 31]. *TelomereAge* was reverse scored such that higher values reflect higher methylation-based age to be consistent with other measures (referred to as *TelomereAgeRev* hereafter).

### Statistical analysis

Reliability (intraclass correlation) was calculated from replicated measurements of methylation-based ageing variables from participants who were included in more than one of the underlying case–control studies and thus provided ≥ 2 measurements. Participants with extreme FFQ estimated energy intake (< 1st quantile or > 99th quantile) were excluded from the analysis. Dietary exposure variables were truncated at the 99th percentile to limit the influence of extreme observations. Dietary exposures were rescaled to units of approximately one standard deviation (rounded to the nearest whole number, where reasonable) to ensure estimates were on a common scale. We present results with methylation-based ageing markers standardised to unit variance (*z* scores) to allow comparability of coefficients (main text) and additionally in their original units (Supplementary Material). Participants with missing data in any of the confounders considered were excluded from the analysis (*N* = 343).

#### Canonical correlation

A preliminary description of the relationship between food group intakes and methylation-based markers of ageing outcomes was obtained using sparse canonical correlation in R package PMA [32]. The method identifies canonical variables *U*, a weighted sum of food group intakes, and *V*, a weighted sum of methylation-based ageing variables, respectively, where weights (canonical coefficients) are estimated to maximise the correlation between the resulting canonical variables. The resulting coefficients identify overlapping patterns in food group intakes and methylation-based markers of ageing and identify the variables that contribute most strongly to the overlap. Prior to fitting the canonical correlation model, ageing outcomes were detrended for age (i.e. the residuals of regressing ageing markers on chronological age were used). To increase the interpretability of the model, small canonical coefficients were shrunk towards zero using the LASSO penalty.

#### Relative and total causal effects using the all-components model

Confounding variables for baseline cross-sectional models were selected according to a directed acyclic graph (eFigure 1). These included age, sex, country of birth, SEIFA decile (neighbourhood socioeconomic status), blood sample type, case–control study, assay slide, smoking status, smoking pack years, educational attainment (ordinal with 8 levels), and score reflecting self-reported physical activity. For the latter, frequency responses (0 [none], 1.5 [one or two times per week], and 4 [≥ 3 times per week]) for walking and less vigorous and vigorous activity were summed after assigning twice the weight to vigorous activity. The overall score ranged from 0 to 16 and was divided into roughly equal groups using the cut points: (0, > 0 to < 4, 4 to < 6, ≥ 6). Continuous confounder’s age, smoking pack years, and alcohol intake were modelled using restricted cubic splines with knots at the 10th, 50th and 90th percentiles. SEIFA decile and education were treated as continuous and linear.

The primary analysis used linear mixed effects regression with adjustment for confounding variables. Random intercepts were included for participants (to account for repeated measures for some participants), case–control study, and assay slide. All dietary exposure variables from a given category, such as macronutrients and subcomponents, were entered into models simultaneously. For instance, the macronutrient and subcomponent model included protein, sugar, starch and dextrins, PUFAs, MUFAs, and saturated fat as exposure terms, along with confounders (total fat and total carbohydrate were not included in this model due to perfect collinearity with their subcomponents). This formulation, in which all dietary exposures from a particular category are included in models together, has been referred to as the ‘all-components’ model [33, 34]. The all-components model allows for estimating ‘total’ and ‘equal-mass substitution causal effects’. Our estimand of primary interest was the ‘equal-mass substitution effect’, which estimates the effect of adding the component of interest to the diet while removing a mass-equivalent amount of the remaining dietary components. Thus, the overall food mass is kept constant (though not necessarily isocaloric). The equal-mass substitution causal effect was estimated by subtracting a weighted average of the coefficients of all other dietary components from the coefficient of interest, where weights were the proportions of remaining total food mass (that is, excluding the exposure of interest) contributed by each component. Confidence intervals for these effect estimates were computed using simulation-based inference [35].

We additionally sought to estimate the ‘total causal effect’, which corresponds to a hypothetical intervention in which a unit of the dietary component of interest is added on top of the current diet, thereby increasing the overall food mass [33, 34]. Estimates of the total causal effect are given directly by the regression coefficient for the component of interest from the all-components model. Associations for dietary quality scores, total energy intake, and coffee and tea intake were each estimated using separate models, with adjustment for the above-described confounding variables. Coffee and tea models were additionally adjusted for total energy intake and dietary quality score (AHEI-2010). Dietary quality models were additionally adjusted for total energy intake (to estimate the effect of an energy-neutral increase in dietary quality). Similarly, to estimate the effect of a quality-neutral increase on total energy, models for total energy intake were adjusted for dietary quality (AHEI-2010).

Data analysis was performed in R version 4.2.2.

## Results

The flow diagram for participant selection in the analysis is displayed in eFigure 2. Characteristics of the sample are provided in Table 1. The final sample included 5310 participants and 5854 measurements of methylation-based markers of ageing. Intraclass correlations for methylation-based markers of ageing were generally moderate to high (range: *DunedinPACE*, 0.70; *PCGrimAge*, 0.89; eTable 2).

**Table 1:** Sample characteristics

Characteristic	Summary
Age	59.2 (7.6)
Male sex, *n* (%)	3590 (68)
Region of birth, *n* (%)	
Australia/New Zealand	3554 (67)
Southern Europe	1409 (27)
Northern Europe	347 (7)
Smoking status, *n* (%)	
Never	2410 (45)
Former	2117 (40)
Current	783 (15)
BMI	27.2 (4.0)
Physical activity category, *n* (%)	
Low	1165 (22)
Medium	3056 (58)
High	1089 (21)
Sample type, *n* (%)	
GC	3822 (72)
Lymph	1399 (26)
BC	89 (2)
PCGrimAge	66 (7.0)
DunedinPACE	0.94 (0.13)
PCPhenoAge	51 (98.9
TelomereAge	6.7 (0.25)
ZhangAge	− 3.1 (0.51)

Summarise are mean (SD) unless otherwise stated. For participants who were included in multiple of the nested-case control studies from which these data are drawn (*n* = 526), only one set of measurements (randomly selected) was included in this table

### Canonical correlation

**Fig. 1:**
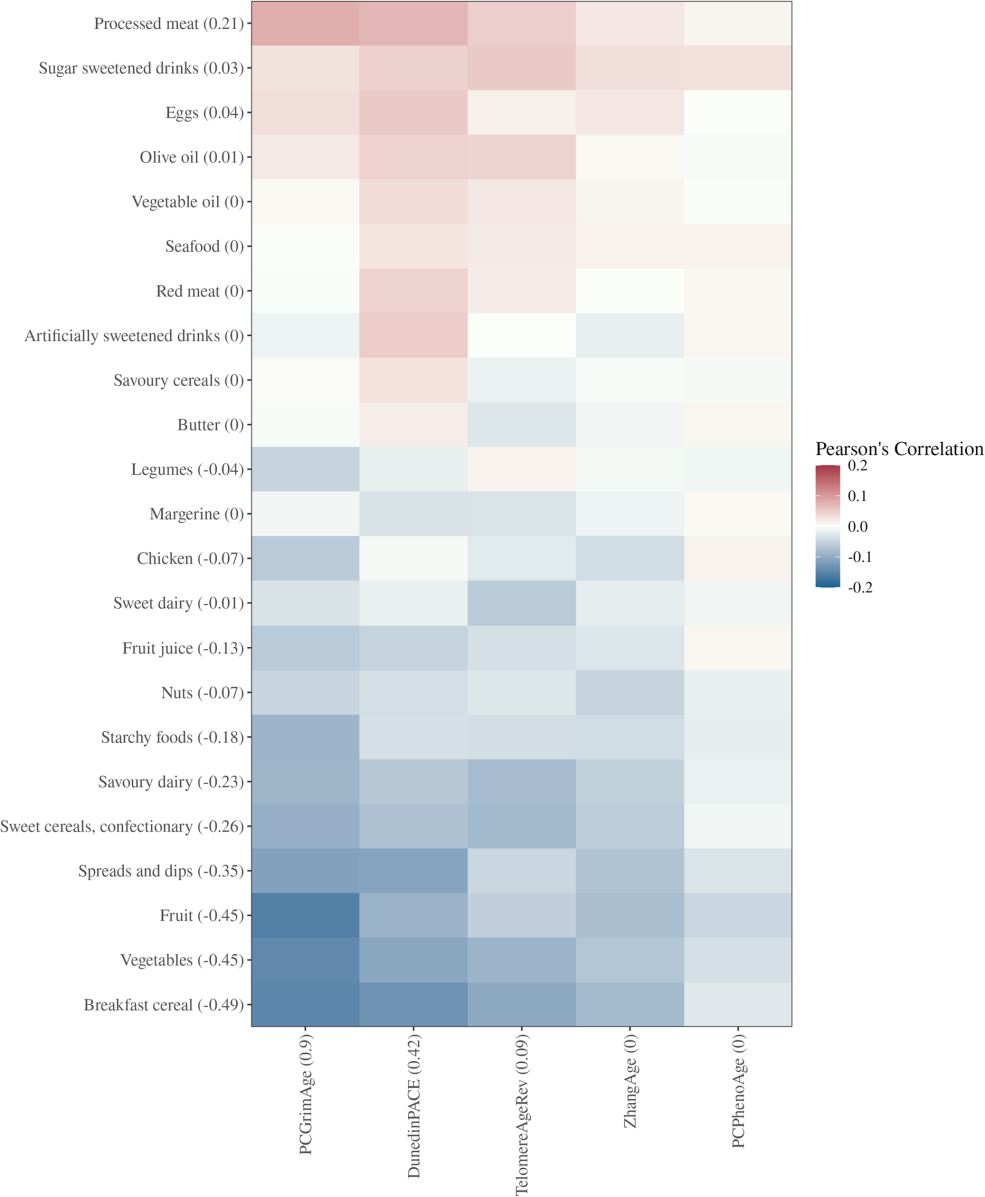
Sparse canonical correlation of food groups and methylation-based markers of ageing. The heat map represents correlation coefficients between individual food group intakes and methylation-based markers of ageing variables. Numbers in parentheses represent canonical coefficients for the food group ‘pattern’ and methylation-based markers of ageing ‘pattern’, respectively. Methylation-based ageing markers have been ‘de-trended’ for age by regressing the marker on chronological age and retaining the residuals

The canonical variables (‘patterns’) estimated by sparse canonical correlation included four methylation-based ageing markers and 17 food groups, respectively (Fig. 1). The correlation of the scores for the two patterns was 0.26. The methylation-based markers of the ageing pattern were formed by elevated *PCGrimAge* and, less strongly, by elevated *DunedinPACE* and *TelomereAgeRev*. The food group pattern consisted of a low intake of breakfast cereal, fruit, vegetables, spreads and dips, sweet cereals and confectionary, savoury dairy products, starchy foods, fruit juice, nuts, chicken, legumes, and sweet dairy products, and high intake of processed meat, eggs, sugar sweetened drinks, and olive oil.

### Macronutrients and sub-components

**Fig. 2:**
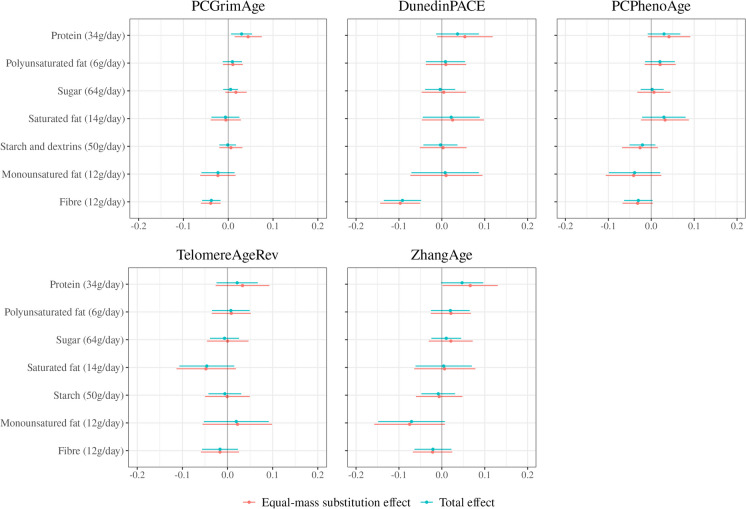
Macronutrients and sub-components and methylation-based markers of ageing. Models included all nutrient exposures and were adjusted for age, sex, country of birth, SEIFA decile, blood sample type, case–control study, assay slide, smoking, smoking pack years, education, and self-reported physical activity score. The equal-mass substitution effect represents the effect of adding a given dietary component to the diet while removing a mass-equivalent amount of the remaining components. The total causal effect represents the effect of adding a given amount of the dietary component to the diet while holding all other components constant. Methylation-based ageing markers are standardised to unit variance

Results from the assessment of associations of macronutrients and their sub-components with methylation-based markers of ageing are presented in Fig. 2. Estimated equal-mass substitution effects and total effect estimates were generally very similar. Considering the former, the strongest inverse associations were observed for fibre intake and *DunedinPACE* (per 12 g/day − 0.10 [standard deviations]; 95%CI − 0.14, − 0.05, *p* < 0.001). Fibre intake was also inversely associated with *PCGrimAge* (per 12 g/day − 0.04; 95%CI − 0.06, − 0.02, *p* < 0.001). Protein intake was positively associated with methylation-based markers of ageing, albeit often weakly (e.g. *PCGrimAge*, per 34 g/day 0.04; 95%CI 0.01, 0.08, *p* = 0.005). The other macronutrients and sub-components had generally weaker, ambiguous associations with the methylation-based markers of ageing.

### Food groups

**Fig. 3:**
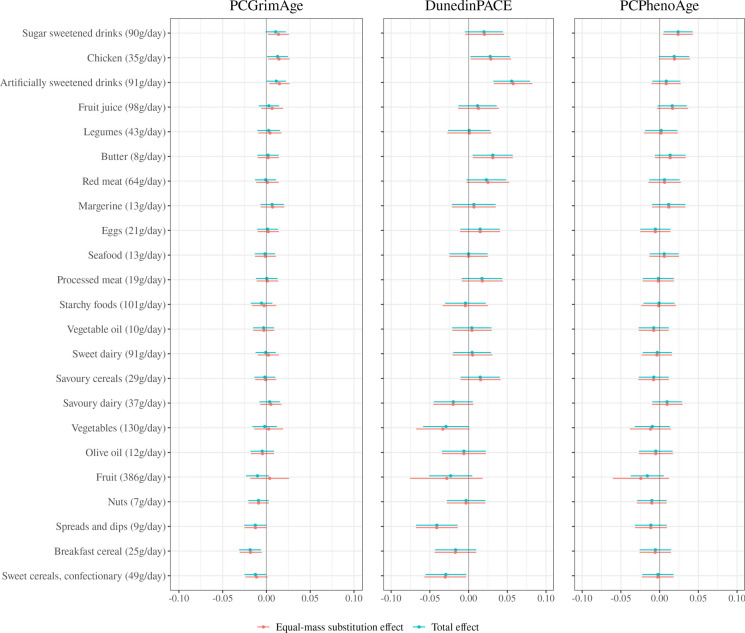

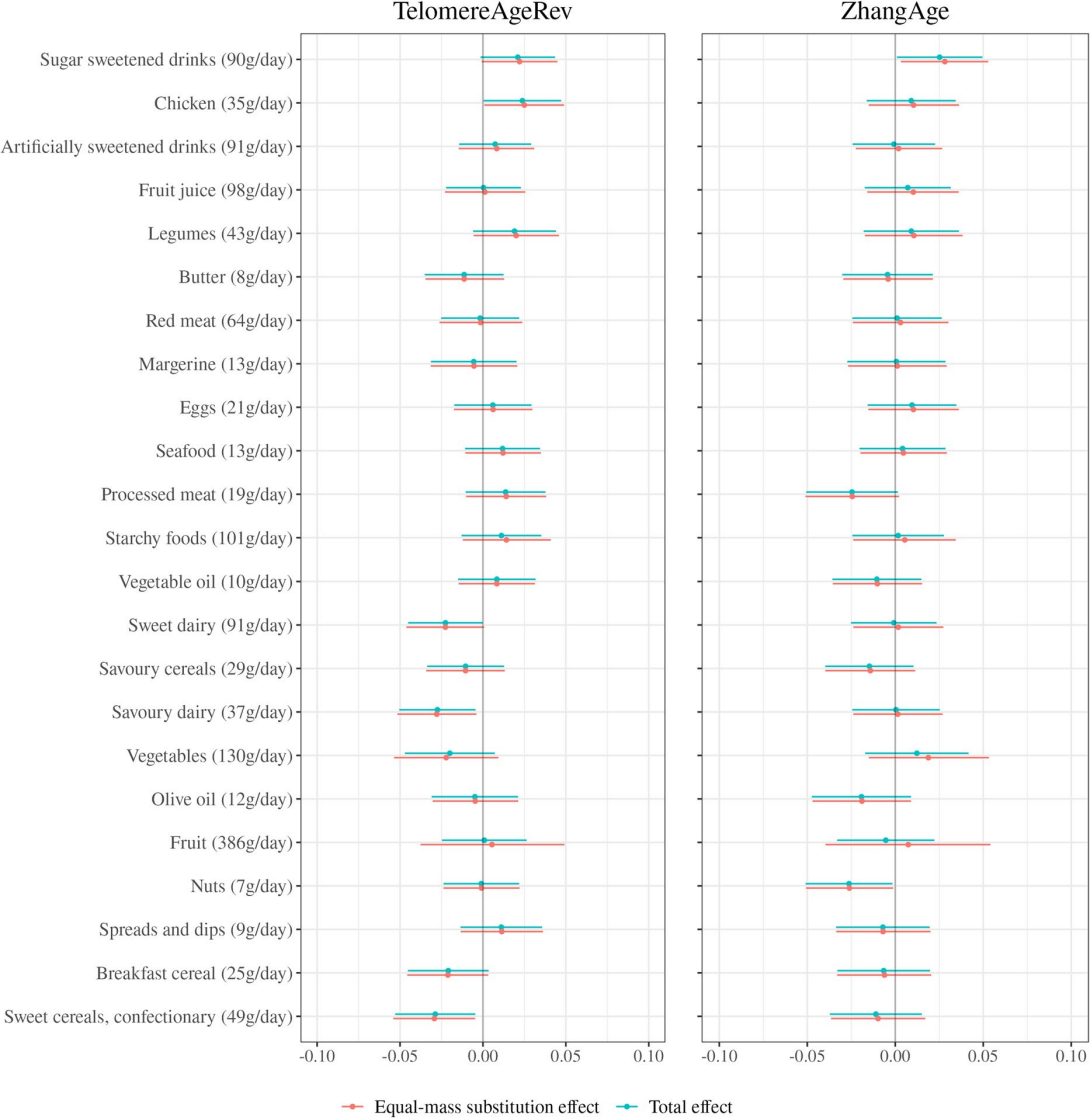
**A** Food groups and methylation-based markers of ageing. Models included all nutrient exposures and were adjusted for age, sex, country of birth, SEIFA decile, blood sample type, case–control study, assay slide, smoking, smoking pack years, education, and self-reported physical activity score. The equal-mass substitution effect represents the effect of adding a given dietary component to the diet while removing a mass-equivalent amount of the remaining components. The total effect represents the effect of adding a given amount of the dietary component to the diet while holding all other components constant. Food groups are rescaled to units of one standard deviation, rounded to the nearest whole number. Methylation-based ageing markers are standardised to unit variance. **B** Food groups and methylation-based markers of ageing. Models included all nutrient exposures and were adjusted for age, sex, country of birth, SEIFA decile, blood sample type, case–control study, assay slide, smoking, smoking pack years, education, and self-reported physical activity score. The equal-mass substitution effect represents the effect of adding a given dietary component to the diet while removing a mass-equivalent amount of the remaining components. The total effect represents the effect of adding a given amount of the dietary component to the diet while holding all other components constant. Food groups are rescaled to units of one standard deviation, rounded to the nearest whole number. Methylation-based ageing markers are standardised to unit variance

Associations between food group intakes and methylation-based markers of ageing are presented in Fig. 3A and B. The equal-mass substitution and total effect estimates were generally in close agreement, though less so in the case of the estimates for fruit intake (the equal-mass substitution effects generally had wider confidence intervals). Considering the equal-mass substitution effects, intake of sugar-sweetened drinks was consistently positively associated with methylation-based ageing markers (e.g. *ZhangAge*, per 90 g/day; 0.03, 95%CI 0.00, 0.05, *p* = 0.026 and *PCPhenoAge*; 0.02, 95%CI 0.01, 0.04, *p* = 0.015). Artificially sweetened drinks also tended to be positively associated with ageing markers, especially with *DunedinPACE* (per 91 g/day 0.06, 95%CI 0.03, 0.08, *p* < 0.001). Associations tended to be negative for ‘sweet cereals and confectionary’ (e.g. *DunedinPACE*, per 49 g/day − 0.03, 95%CI − 0.06, − 0.00, *p* = 0.029) and breakfast cereals (e.g. *PCGrimAge*, per 25 g/day − 0.02, 95%CI − 0.03, − 0.01, *p* = 0.003). Associations for other food groups were weaker and ambiguous.

### Dietary quality

**Fig. 4:**
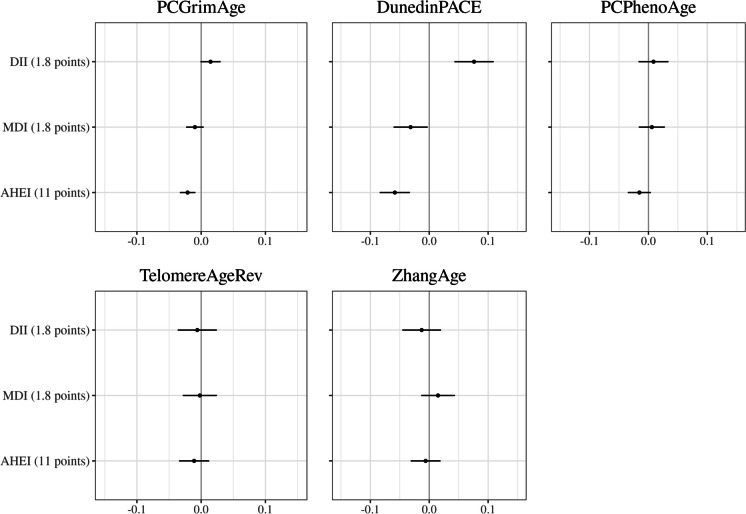
Dietary quality and methylation-based markers of ageing. Models were fitted individually for each dietary quality score and were adjusted for age, sex, country of birth, SEIFA decile, blood sample type, case–control study, assay slide, smoking, smoking pack years, education, self-reported physical activity score, and total energy intake. Methylation-based ageing markers are standardised to unit variance

The associations between dietary quality indices and methylation-based markers of ageing are presented in Fig. 4. AHEI was inversely associated with *DunedinPACE* (per 11 points − 0.06; 95%CI − 0.08, − 0.03, *p* < 0.001) and *PCGrimAge* (per 11 points − 0.02, 95%CI − 0.03, − 0.01, *p* < 0.001) whereas associations for MDI were weaker and more ambiguous. DII^©^ was positively associated with *DunedinPACE* (per 1.8 points 0.08; 95%CI 0.04, 0.11, *p* < 0.001), and associations with other ageing markers tended to be weaker.

### Other dietary factors

**Fig. 5:**
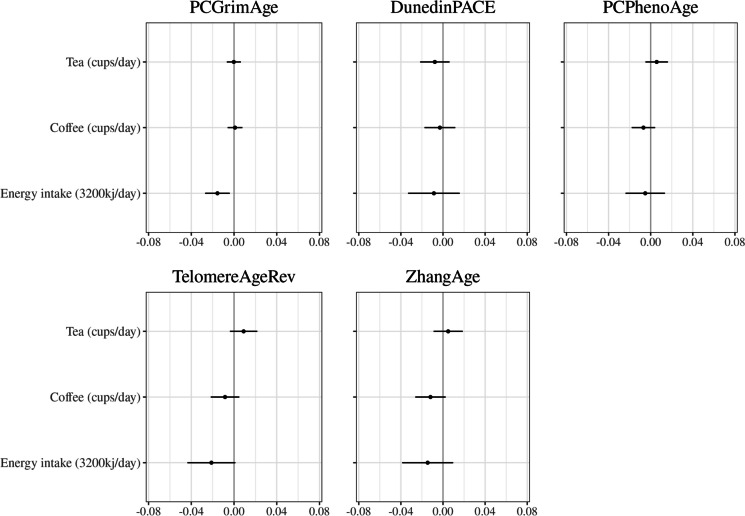
Coffee, tea, and total energy intake and methylation-based markers of ageing. Models were fitted individually for each of tea, coffee, and energy intake and were adjusted for age, sex, country of birth, SEIFA decile, blood sample type, case–control study, assay slide, smoking, smoking pack years, education, and self-reported physical activity score. Models for coffee and tea are additionally adjusted for dietary quality (AHEI-2010) and total energy intake. Models for total energy intake are additionally adjusted for dietary quality score (AHEI-2010). Methylation-based ageing markers are standardised to unit variance

The associations between total energy intake and coffee and tea intake and methylation-based markers of ageing are shown in Fig. 5. Energy intake was inversely associated with *PCGrimAge* (per 3200 kJ − 0.03, 95%CI − 0.05, − 0.01, *p* = 0.008). Other estimates were generally small and ambiguous.

Equivalent results are presented with methylation-based ageing markers in their original units in the Supplementary Material (eFigures 3–6).

## Discussion

We investigated the cross-sectional associations of dietary composition, including macronutrients, food groups, and overall diet quality, with several methylation-based markers of ageing. First, *PCGrimAge* and *DunedinPACE* were generally more consistently associated with dietary variables than were the other biological ageing markers. Second, higher dietary quality was associated with reduced methylation-based markers of ageing, at least for *PCGrimAge* and *DunedinPACE*. Third, considering macronutrients, we found higher fibre intake to be associated with reduced methylation-based markers of ageing while greater protein intake was associated with increased ageing markers, and energy intake was inversely associated with *PCGrimAge*. Finally, despite suggestive evidence for some food groups (e.g. a positive association for sugar- and artificially sweetened drinks), the contribution of individual food groups/items to methylation-based markers of ageing appeared to be generally small or null.

One limitation of the present study is that, due to the cross-sectional design, it is not clear when participants began certain dietary habits or for how long they had been maintained prior to study entry. Accordingly, the estimated associations cannot be interpreted with respect to clearly defined hypothetical dietary interventions (which would require the characterisation of the time of initiation and duration that the dietary intervention was maintained) [36]. Measurement error is also a source of bias. While our FFQ was designed specifically for the MCCS cohort, based on weighed food records in similar people [37], significant measurement error is likely to remain, and this would also introduce bias to effect estimates, generally towards the null. The biological ageing measures were similarly imperfectly measured, though their reliabilities were estimated to be very good to excellent (all ICC ≥ 0.70), which would generally also introduce a bias generally towards the null. Lastly, as an observational study, we cannot rule out the presence of residual confounding, for instance, due to incomplete adjustment for SES (education and neighbourhood SES are imperfect proxies) or other unmeasured common causes of dietary habits and methylation-based ageing. One potential source of residual confounding is physical health and comorbidity. It is possible that people in ill health, for example, whether diagnosed with a specific condition or not, may have adopted healthier dietary habits, which would obscure the beneficial effect of healthy foods on epigenetic ageing. Rich data on health and comorbidity was not available to adjust for such factors in this analysis. Nevertheless, while adjusting for these variables may have reduced residual confounding, it may also have introduced some ‘overadjustment bias’ [38], as health and disease is a plausible mechanism through which diet would influence biological ageing outcomes (i.e. these factors may be on the causal path between diet and biological ageing). Finally, our aggregation of foods into food groups and nutrients into overall macronutrient summaries may have masked heterogeneity within these aggregate variables. For instance, while we found that greater protein intake was associated with advanced epigenetic age, this may plausibly differ between protein sources derived from red meat sources and those from fish or poultry, for example [39].

The main strengths of this study include (i) the use of the most recent and widely used measures of methylation-based markers of ageing: many studies have focused on first-generation markers, which show very weak associations with disease and mortality risk [40], and to our knowledge, virtually no studies have considered telomere length or the Zhang predictor of mortality, or the improved versions of *GrimAge* or *PhenoAge* based on principal components; we acknowledge that additional measures such as *bAge* [37], *DNAmFitAge* [41], *GrimAge2* [42], or the individual components of *GrimAge* could be investigated in future studies; (ii) our sample size of > 5300 participants (> 5800 samples), which is much greater than most studies in the field; (iii) the extensive range of dietary factors we considered; and (iv) the use of methods to estimate clear target parameters, namely the equal-mass substitution effect and the total causal effect [33, 34].

The study by Fitzgerald et al. [12] demonstrates the proof of concept that lifestyle interventions can lower epigenetic age but, because the intervention they implemented was multimodal, it was not possible to isolate the specific effect of diet. The diet promoted in this trial focused on animal protein and avoided grain and legumes, so would have missed important sources of fibre. It should be noted that this trial’s endpoint was Horvath’s first-generation epigenetic clock, which we did not consider here, and only included 38 men (18 in the treatment group). The DAMA study [13] included 219 women divided into four intervention groups and reported a positive effect of a 2-year diet intervention on slowing down biological ageing as assessed by *GrimAge*. Although their dietary intervention was complex, it likely resulted in substantially increased fibre intake and some decrease in protein intake and this result is therefore broadly consistent with our key findings at the level of macronutrients. It is unclear, however, whether women lost weight or consumed less energy during the intervention, which would have an influence on methylation-based ageing markers and limit the direct comparability with our estimates. The baseline cross-sectional analysis showed intakes of fruit and vegetables to be the only specific dietary factors negatively associated with *GrimAge* [13]. In our study, the coefficients for fruit and vegetable intakes were generally in the expected direction, though confidence intervals often contained the null. A post hoc analysis of the Comprehensive Assessment of Long-term Effects of Reducing Intake of Energy (CALERIE) randomised controlled trial in which adults without obesity were randomised to 25% caloric restriction (*N* = 128) or ad libitum control diet for 2 years (*N* = 69) found that caloric restriction resulted in lower *DunedinPACE*, but not *GrimAge* and *PhenoAge* [43]. These results were not consistent with our study since we found a negative association for *GrimAge* and energy intake and null associations for *DunedinPACE* and *PhenoAge*, though this could be attributed to residual confounding (e.g. incomplete adjustment for physical activity) or measurement error.

In conclusion, we found that certain aspects of diet were associated with methylation-based ageing markers in a large cross-sectional study of middle-aged and older adults. Overall, healthy diets and high intakes of fibre and low intakes of proteins were most strongly associated with epigenetic ageing markers. Results consistent with existing knowledge were obtained for several other dietary factors such as sweetened drinks. While existing evidence points to the potential of diet to reduce biological ageing, additional intervention studies and prospective observational studies investigating different aspects of diet, as our study did, would help identify the most important elements of diet for healthy ageing. Studies with long-term follow-up to determine whether diet-related changes in methylation-based markers of ageing affect future health outcomes would also be informative.

## Supplementary Information

Below is the link to the electronic supplementary material.

Supplementary file1 (DOCX 597 KB)

## Data Availability

For participants who provided consent, the methylation data are available under controlled-access at dbGaP (#phs003213.v1.p1, for which more details can be found at https://www.ncbi.nlm.nih.gov/projects/gap/cgi-bin/study.cgi?study_id=phs003213.v1.p1).
