# Supplementary material for: Dietary factors and DNA methylation-based markers of ageing in 5310 middle-aged and older Australian adults
Source: GeroScience. 2024 Sep 19;47(2):1685–98. doi: 10.1007/s11357-024-01341-7 (PMC11978581; doi:10.1007/s11357-024-01341-7)
Supplement: Supplementary file 1 — Supplementary file1 (DOCX 597 KB) [file 11357_2024_1341_MOESM1_ESM.docx]

**eTable 1:** Food groups and constituents

| ***Food group*** | ***Constituents*** | ***Median food group intake (grams) (25^th^-75^th^ percentile)*** | ***Mean proportion of total food mass*** |
| --- | --- | --- | --- |
| Artificially sweetened drinks | Diet (low-cal) soft drink | 0 (0, 0) | 2% |
| Breakfast cereal | Wheat germ, muesli, other breakfast cereals | 24 (2, 40) | 2% |
| Butter | Butter | 0 (0, 1.4) | <1% |
| Chicken | Chicken roast or fried, chicken boiled or steamed, mixed dishes with chicken | 31 (18, 52) | 3% |
| Egg | Egg boiled, egg fried, egg in mixed dishes | 13 (4, 26) | 1% |
| Fruit juice | Orange or lemon juice, other fruit juice | 41 (9, 140) | 5% |
| Fruit | Dried apricots or peaches, fruit salad, oranges or mandarins, apples, bananas, peaches or nectarines, pears, cantaloupe or honeydew melon, watermelon, strawberries, plums, apricots, grapefruit, pineapple, avocado, olives, figs, grapes | 361 (199, 617) | 31% |
| Legumes | Beans, pea or lentil soup, green beans, or peas, cooked dried bean or chickpea dishes | 35 (22, 51) | 3% |
| Margarine | Margarine | 8 (1, 25) | <1% |
| Nuts | Peanuts or peanut butter, other nuts | 2 (0, 5) | <1% |
| Processed meat | Salami or continental sausages, sausages or frankfurters, bacon, ham, corned beef (silverside), manufactured luncheon meats | 16 (7, 28) | 1% |
| Red meat | Veal or beef schnitzel, beef or veal roast, beef steak, rissoles or meatloaf, mixed dishes with beef, lamb chops or lamb roast, mixed dishes with lamb, pork chops or pork roast, rabbit or other game, offal, other offal meats | 82 (52, 122) | 6% |
| Savoury cereals | Dry biscuits, pizza, dim sims or spring rolls, pies or savoury pastries, corn chips or potato chips | 23 (9, 42) | 2% |
| Savoury dairy | Cottage cheese, ricotta cheese, feta cheese, low fat or low cholesterol cheese, hard grating cheese, cream cheese, cheddar or similar cheeses, cream or sour cream, yoghurt | 23 (11, 49) | 2% |
| Seafood | Fish steamed, grilled or baked, fish fried, fish smoked, canned fish, seafood | 10 (5, 19) | <1% |
| Spreads and dips | Dip, jam honey or syrups, vegemite | 5 (1, 12) | <1% |
| Starchy foods | Boiled rice (including brown rice), fried rice, mixed rice dishes, white bread, rolls or toast, wholewheat or rye bread, rolls or toast, fruit bread, pasta and noodles, potato fried or roasted, potato cooked without fat | 203 (143, 270) | 14% |
| Sugar-sweetened drinks | Soft drink | 0 (0, 22) | 3% |
| Sweet cereals, confectionary | Chocolate confectionary, other confectionary, sweet biscuits, cakes or sweet pastries, puddings | 26 (9, 61) | 3% |
| Sweet dairy | Ice cream, custard, milk drinks | 17 (3, 53) | 3% |
| Vegetables | Cabbage and brussels sprouts, cauliflower, broccoli, beetroot, carrot, lettuce, endive or other salad greens, silverbeet, spinach or other leafy greens, onions and leeks, cucumber, zucchini, squash or eggplant, pumpkin, pickled vegetables, capsicum, celery or fennel, coleslaw, mushrooms, sweet corn, mixed vegetable dishes, creamed soups, other soups or broths | 191 (121, 281) | 14% |

**eTable 2.** Intraclass correlations of methylation-based ageing markers

| Outcome | Intraclass correlation |
| --- | --- |
| *PCGrimAge* | 0.89 |
| *PCPhenoAge* | 0.79 |
| *ZhangAge* | 0.71 |
| *DunedinPACE* | 0.70 |
| *TelomereAgeRev* | 0.73 |

Intraclass correlations were calculated from participants with replicated observations (n = 526, providing 1,086 total measurements) by dividing between-subject variance by the sum of between-subject and within-subject variance, conditional on chronological age.

**eTable 3**. Macronutrients and sub-components and methylation-based markers of ageing*

| Exposure | PCGrimAge | PCPhenoAge | ZhangAge | TelomereAgeRev | DunedinPACE |
| --- | --- | --- | --- | --- | --- |
| *Relative equal mass effect* | | | | | |
| Protein (34g/day) | 0.044 (0.013,0.075), p=0.005 | 0.041 (-0.009,0.090), p=0.104 | 0.066 (0.002,0.130), p=0.042 | 0.033 (-0.028,0.092), p=0.282 | 0.053 (-0.011,0.118), p=0.105 |
| Sugar (64g/day) | 0.017 (-0.007,0.041), p=0.162 | 0.006 (-0.032,0.045), p=0.747 | 0.021 (-0.030,0.071), p=0.417 | 0.000 (-0.047,0.047), p=0.995 | 0.005 (-0.046,0.056), p=0.855 |
| Starch (50g/day) | 0.006 (-0.020,0.032), p=0.649 | -0.026 (-0.067,0.016), p=0.224 | -0.006 (-0.060,0.048), p=0.824 | -0.001 (-0.051,0.050), p=0.972 | 0.003 (-0.051,0.058), p=0.911 |
| Fibre (12g/day) | -0.039 (-0.061,-0.018), p<0.001 | -0.032 (-0.067,0.003), p=0.078 | -0.021 (-0.067,0.025), p=0.364 | -0.017 (-0.059,0.026), p=0.444 | -0.097 (-0.143,-0.051), p<0.001 |
| Saturated fat (14g/day) | -0.005 (-0.039,0.029), p=0.777 | 0.032 (-0.023,0.087), p=0.256 | 0.006 (-0.064,0.078), p=0.870 | -0.048 (-0.114,0.017), p=0.148 | 0.025 (-0.046,0.097), p=0.493 |
| Monounsaturated fat (12g/day) | -0.023 (-0.063,0.016), p=0.258 | -0.041 (-0.104,0.024), p=0.209 | -0.075 (-0.158,0.008), p=0.076 | 0.022 (-0.055,0.100), p=0.576 | 0.009 (-0.074,0.094), p=0.827 |
| Polyunsaturated fat (6g/day) | 0.010 (-0.012,0.032), p=0.363 | 0.021 (-0.015,0.057), p=0.253 | 0.021 (-0.025,0.067), p=0.381 | 0.008 (-0.034,0.051), p=0.704 | 0.009 (-0.038,0.056), p=0.696 |
| *Total effect* | | | | | |
| Protein (34g/day) | 0.030 (0.006,0.053), p=0.013 | 0.030 (-0.008,0.068), p=0.126 | 0.047 (-0.002,0.097), p=0.061 | 0.021 (-0.024,0.067), p=0.359 | 0.037 (-0.013,0.087), p=0.150 |
| Sugar (64g/day) | 0.005 (-0.011,0.022), p=0.531 | 0.002 (-0.025,0.029), p=0.878 | 0.010 (-0.024,0.045), p=0.562 | -0.007 (-0.039,0.025), p=0.685 | -0.004 (-0.039,0.031), p=0.827 |
| Starch (50g/day) | -0.001 (-0.020,0.018), p=0.903 | -0.020 (-0.051,0.010), p=0.186 | -0.008 (-0.048,0.031), p=0.678 | -0.006 (-0.042,0.030), p=0.750 | -0.003 (-0.043,0.036), p=0.873 |
| Fibre (12g/day) | -0.038 (-0.058,-0.017), p<0.001 | -0.030 (-0.063,0.003), p=0.076 | -0.021 (-0.064,0.022), p=0.345 | -0.017 (-0.057,0.023), p=0.412 | -0.092 (-0.135,-0.048), p<0.001 |
| Saturated fat (14g/day) | -0.006 (-0.037,0.025), p=0.695 | 0.029 (-0.021,0.080), p=0.254 | 0.004 (-0.061,0.070), p=0.897 | -0.046 (-0.107,0.015), p=0.139 | 0.022 (-0.045,0.088), p=0.521 |
| Monounsaturated fat (12g/day) | -0.023 (-0.060,0.014), p=0.226 | -0.039 (-0.099,0.021), p=0.204 | -0.071 (-0.148,0.007), p=0.075 | 0.019 (-0.053,0.091), p=0.597 | 0.008 (-0.071,0.086), p=0.848 |
| Polyunsaturated fat (6g/day) | 0.009 (-0.012,0.031), p=0.397 | 0.020 (-0.015,0.055), p=0.256 | 0.020 (-0.025,0.065), p=0.391 | 0.007 (-0.034,0.049), p=0.728 | 0.008 (-0.037,0.054), p=0.718 |

* Estimate (95% confidence interval), p value.

Models included all nutrient exposures and were adjusted for age, sex, country of birth, SEIFA decile, blood sample type, case-control study, assay slide, smoking, smoking pack years, education, and self-reported physical activity score. The equal-mass substitution effect represents the effect of adding a given dietary component to the diet while removing a mass-equivalent amount of the remaining components. The total causal effect represents the effect of adding a given amount of the dietary component to the diet while holding all other components constant. Methylation-based ageing markers are standardised to unit variance.

**eTable 4.** Food groups and methylation-based markers of ageing*

| Exposure | PCGrimAge | PCPhenoAge | ZhangAge | TelomereAgeRev | DunedinPACE |
| --- | --- | --- | --- | --- | --- |
| *Relative equal mass effect* | | | | | |
| Breakfast cereal (25g/day) | -0.018 (-0.031,-0.005), p=0.005 | -0.006 (-0.026,0.015), p=0.581 | -0.006 (-0.033,0.020), p=0.653 | -0.021 (-0.046,0.003), p=0.090 | -0.017 (-0.044,0.010), p=0.215 |
| Sweet cereals, confectionary (49g/day) | -0.011 (-0.024,0.001), p=0.077 | -0.002 (-0.023,0.018), p=0.817 | -0.010 (-0.036,0.017), p=0.474 | -0.029 (-0.054,-0.005), p=0.020 | -0.030 (-0.057,-0.003), p=0.029 |
| Savoury cereals (29g/day) | -0.001 (-0.013,0.011), p=0.879 | -0.008 (-0.027,0.012), p=0.438 | -0.014 (-0.040,0.011), p=0.278 | -0.010 (-0.034,0.013), p=0.387 | 0.015 (-0.010,0.041), p=0.242 |
| Starchy foods (101g/day) | -0.003 (-0.017,0.011), p=0.708 | -0.001 (-0.024,0.021), p=0.907 | 0.005 (-0.024,0.034), p=0.716 | 0.014 (-0.012,0.041), p=0.295 | -0.004 (-0.034,0.025), p=0.775 |
| Savoury dairy (37g/day) | 0.005 (-0.007,0.017), p=0.422 | 0.010 (-0.010,0.030), p=0.332 | 0.001 (-0.024,0.027), p=0.912 | -0.028 (-0.052,-0.004), p=0.022 | -0.020 (-0.046,0.006), p=0.131 |
| Sweet dairy (91g/day) | 0.002 (-0.010,0.014), p=0.718 | -0.004 (-0.023,0.016), p=0.726 | 0.002 (-0.024,0.027), p=0.895 | -0.023 (-0.046,0.001), p=0.059 | 0.005 (-0.021,0.031), p=0.698 |
| Eggs (21g/day) | 0.002 (-0.010,0.014), p=0.738 | -0.006 (-0.025,0.014), p=0.578 | 0.010 (-0.015,0.036), p=0.433 | 0.006 (-0.017,0.030), p=0.610 | 0.015 (-0.011,0.041), p=0.259 |
| Butter (8g/day) | 0.002 (-0.010,0.014), p=0.746 | 0.014 (-0.006,0.034), p=0.181 | -0.004 (-0.030,0.021), p=0.758 | -0.011 (-0.035,0.013), p=0.352 | 0.031 (0.006,0.057), p=0.017 |
| Margerine (13g/day) | 0.007 (-0.007,0.020), p=0.314 | 0.012 (-0.010,0.033), p=0.283 | 0.001 (-0.027,0.029), p=0.936 | -0.005 (-0.031,0.021), p=0.685 | 0.007 (-0.021,0.035), p=0.631 |
| Red meat (64g/day) | 0.001 (-0.012,0.014), p=0.852 | 0.006 (-0.014,0.027), p=0.548 | 0.003 (-0.024,0.030), p=0.829 | -0.001 (-0.026,0.024), p=0.912 | 0.025 (-0.002,0.052), p=0.074 |
| Processed meat (19g/day) | 0.001 (-0.011,0.013), p=0.876 | -0.002 (-0.022,0.018), p=0.870 | -0.024 (-0.051,0.002), p=0.072 | 0.014 (-0.010,0.038), p=0.255 | 0.018 (-0.009,0.044), p=0.190 |
| Chicken (35g/day) | 0.014 (0.002,0.027), p=0.023 | 0.019 (-0.001,0.039), p=0.058 | 0.010 (-0.015,0.036), p=0.426 | 0.025 (0.001,0.049), p=0.042 | 0.029 (0.003,0.055), p=0.031 |
| Seafood (13g/day) | -0.001 (-0.013,0.011), p=0.836 | 0.006 (-0.013,0.025), p=0.543 | 0.005 (-0.020,0.029), p=0.711 | 0.012 (-0.011,0.035), p=0.297 | -0.000 (-0.025,0.025), p=1.000 |
| Vegetables (130g/day) | 0.003 (-0.014,0.019), p=0.757 | -0.012 (-0.038,0.015), p=0.388 | 0.019 (-0.015,0.053), p=0.280 | -0.022 (-0.054,0.009), p=0.167 | -0.033 (-0.068,0.001), p=0.059 |
| Fruit (386g/day) | 0.004 (-0.018,0.026), p=0.744 | -0.024 (-0.060,0.012), p=0.188 | 0.007 (-0.040,0.054), p=0.757 | 0.005 (-0.038,0.049), p=0.806 | -0.028 (-0.076,0.018), p=0.239 |
| Legumes (43g/day) | 0.004 (-0.009,0.017), p=0.550 | 0.002 (-0.020,0.023), p=0.864 | 0.011 (-0.017,0.038), p=0.455 | 0.020 (-0.006,0.046), p=0.127 | 0.001 (-0.027,0.029), p=0.944 |
| Fruit juice (98g/day) | 0.006 (-0.006,0.019), p=0.311 | 0.017 (-0.003,0.037), p=0.103 | 0.010 (-0.016,0.036), p=0.440 | 0.001 (-0.023,0.026), p=0.923 | 0.013 (-0.013,0.039), p=0.343 |
| Artificially sweetened drinks (91g/day) | 0.015 (0.003,0.026), p=0.012 | 0.009 (-0.010,0.027), p=0.375 | 0.002 (-0.022,0.027), p=0.880 | 0.008 (-0.015,0.031), p=0.474 | 0.058 (0.033,0.082), p<0.001 |
| Sugar sweetened drinks (90g/day) | 0.014 (0.002,0.026), p=0.023 | 0.024 (0.005,0.043), p=0.015 | 0.028 (0.003,0.053), p=0.026 | 0.022 (-0.001,0.045), p=0.058 | 0.021 (-0.004,0.046), p=0.109 |
| Nuts (7g/day) | -0.009 (-0.021,0.003), p=0.131 | -0.010 (-0.029,0.009), p=0.304 | -0.026 (-0.051,-0.001), p=0.039 | -0.001 (-0.024,0.022), p=0.942 | -0.003 (-0.028,0.022), p=0.796 |
| Spreads and dips (9g/day) | -0.013 (-0.025,0.000), p=0.050 | -0.011 (-0.032,0.009), p=0.279 | -0.007 (-0.034,0.020), p=0.612 | 0.011 (-0.013,0.036), p=0.370 | -0.041 (-0.068,-0.014), p=0.003 |
| Olive oil (12g/day) | -0.004 (-0.018,0.009), p=0.507 | -0.005 (-0.027,0.017), p=0.656 | -0.019 (-0.047,0.009), p=0.185 | -0.005 (-0.030,0.021), p=0.723 | -0.006 (-0.034,0.022), p=0.676 |
| Vegetable oil (10g/day) | -0.003 (-0.015,0.009), p=0.629 | -0.008 (-0.027,0.012), p=0.439 | -0.010 (-0.036,0.015), p=0.431 | 0.008 (-0.015,0.031), p=0.476 | 0.004 (-0.021,0.030), p=0.738 |
|  | | | | | |
| *Total effect* | | | | | |
| Breakfast cereal (25g/day) | -0.019 (-0.031,-0.006), p=0.003 | -0.005 (-0.026,0.015), p=0.600 | -0.007 (-0.033,0.020), p=0.625 | -0.021 (-0.045,0.003), p=0.092 | -0.017 (-0.043,0.010), p=0.217 |
| Sweet cereals, confectionary (49g/day) | -0.013 (-0.025,-0.000), p=0.045 | -0.002 (-0.022,0.018), p=0.830 | -0.011 (-0.037,0.015), p=0.409 | -0.029 (-0.053,-0.005), p=0.020 | -0.029 (-0.056,-0.003), p=0.029 |
| Savoury cereals (29g/day) | -0.002 (-0.014,0.010), p=0.750 | -0.007 (-0.027,0.012), p=0.447 | -0.015 (-0.040,0.010), p=0.251 | -0.010 (-0.034,0.013), p=0.377 | 0.015 (-0.010,0.040), p=0.244 |
| Starchy foods (101g/day) | -0.006 (-0.018,0.007), p=0.370 | -0.001 (-0.021,0.019), p=0.942 | 0.002 (-0.024,0.028), p=0.900 | 0.011 (-0.013,0.035), p=0.363 | -0.004 (-0.030,0.022), p=0.765 |
| Savoury dairy (37g/day) | 0.004 (-0.008,0.015), p=0.549 | 0.010 (-0.010,0.029), p=0.325 | 0.000 (-0.024,0.025), p=0.972 | -0.027 (-0.050,-0.004), p=0.019 | -0.020 (-0.045,0.005), p=0.124 |
| Sweet dairy (91g/day) | -0.001 (-0.012,0.011), p=0.878 | -0.003 (-0.022,0.016), p=0.756 | -0.001 (-0.025,0.023), p=0.945 | -0.023 (-0.045,-0.000), p=0.049 | 0.005 (-0.020,0.029), p=0.715 |
| Eggs (21g/day) | 0.001 (-0.011,0.013), p=0.831 | -0.005 (-0.025,0.014), p=0.583 | 0.010 (-0.016,0.035), p=0.459 | 0.006 (-0.017,0.029), p=0.614 | 0.015 (-0.011,0.040), p=0.251 |
| Butter (8g/day) | 0.002 (-0.010,0.014), p=0.774 | 0.014 (-0.006,0.033), p=0.179 | -0.004 (-0.030,0.021), p=0.739 | -0.011 (-0.035,0.012), p=0.350 | 0.031 (0.005,0.057), p=0.018 |
| Margerine (13g/day) | 0.006 (-0.007,0.020), p=0.340 | 0.012 (-0.010,0.033), p=0.285 | 0.001 (-0.027,0.029), p=0.963 | -0.006 (-0.031,0.020), p=0.673 | 0.007 (-0.021,0.035), p=0.637 |
| Red meat (64g/day) | -0.001 (-0.013,0.011), p=0.876 | 0.006 (-0.013,0.026), p=0.533 | 0.001 (-0.024,0.026), p=0.936 | -0.002 (-0.025,0.022), p=0.889 | 0.023 (-0.003,0.049), p=0.080 |
| Processed meat (19g/day) | 0.000 (-0.012,0.013), p=0.956 | -0.002 (-0.022,0.018), p=0.872 | -0.025 (-0.051,0.001), p=0.065 | 0.014 (-0.010,0.038), p=0.265 | 0.017 (-0.009,0.043), p=0.197 |
| Chicken (35g/day) | 0.013 (0.001,0.025), p=0.038 | 0.019 (-0.001,0.038), p=0.059 | 0.009 (-0.016,0.034), p=0.482 | 0.024 (0.000,0.047), p=0.046 | 0.028 (0.002,0.053), p=0.032 |
| Seafood (13g/day) | -0.002 (-0.013,0.010), p=0.770 | 0.006 (-0.013,0.025), p=0.536 | 0.004 (-0.020,0.029), p=0.739 | 0.012 (-0.011,0.034), p=0.304 | -0.000 (-0.025,0.025), p=0.999 |
| Vegetables (130g/day) | -0.002 (-0.016,0.012), p=0.768 | -0.009 (-0.032,0.013), p=0.418 | 0.012 (-0.017,0.042), p=0.412 | -0.020 (-0.047,0.007), p=0.150 | -0.029 (-0.059,0.000), p=0.054 |
| Fruit (386g/day) | -0.010 (-0.023,0.003), p=0.124 | -0.016 (-0.037,0.005), p=0.144 | -0.005 (-0.033,0.022), p=0.703 | 0.001 (-0.025,0.026), p=0.950 | -0.023 (-0.051,0.005), p=0.104 |
| Legumes (43g/day) | 0.002 (-0.010,0.015), p=0.708 | 0.002 (-0.019,0.023), p=0.846 | 0.009 (-0.018,0.036), p=0.509 | 0.019 (-0.006,0.044), p=0.135 | 0.001 (-0.027,0.028), p=0.955 |
| Fruit juice (98g/day) | 0.003 (-0.009,0.014), p=0.637 | 0.016 (-0.003,0.035), p=0.091 | 0.007 (-0.017,0.032), p=0.568 | 0.000 (-0.022,0.023), p=0.977 | 0.012 (-0.013,0.036), p=0.358 |
| Artificially sweetened drinks (91g/day) | 0.011 (0.000,0.022), p=0.048 | 0.009 (-0.009,0.027), p=0.349 | -0.001 (-0.024,0.023), p=0.947 | 0.007 (-0.014,0.029), p=0.506 | 0.056 (0.032,0.079), p<0.001 |
| Sugar sweetened drinks (90g/day) | 0.011 (-0.001,0.022), p=0.075 | 0.024 (0.005,0.043), p=0.012 | 0.025 (0.001,0.050), p=0.042 | 0.021 (-0.001,0.044), p=0.067 | 0.020 (-0.005,0.044), p=0.113 |
| Nuts (7g/day) | -0.009 (-0.021,0.003), p=0.127 | -0.010 (-0.029,0.009), p=0.301 | -0.026 (-0.051,-0.002), p=0.037 | -0.001 (-0.024,0.022), p=0.934 | -0.003 (-0.028,0.022), p=0.797 |
| Spreads and dips (9g/day) | -0.013 (-0.025,-0.000), p=0.047 | -0.011 (-0.032,0.009), p=0.281 | -0.007 (-0.034,0.020), p=0.604 | 0.011 (-0.014,0.036), p=0.377 | -0.041 (-0.068,-0.014), p=0.003 |
| Olive oil (12g/day) | -0.005 (-0.018,0.009), p=0.477 | -0.005 (-0.027,0.017), p=0.660 | -0.019 (-0.047,0.009), p=0.182 | -0.005 (-0.031,0.021), p=0.715 | -0.006 (-0.034,0.022), p=0.676 |
| Vegetable oil (10g/day) | -0.003 (-0.015,0.009), p=0.599 | -0.008 (-0.027,0.012), p=0.446 | -0.010 (-0.036,0.015), p=0.418 | 0.008 (-0.015,0.032), p=0.483 | 0.004 (-0.021,0.030), p=0.749 |

* Estimate (95% confidence interval), p value.

Models included all nutrient exposures and were adjusted for age, sex, country of birth, SEIFA decile, blood sample type, case-control study, assay slide, smoking, smoking pack years, education, and self-reported physical activity score. Food groups are rescaled to units of one standard deviation, rounded to the nearest whole number. Methylation-based ageing markers are standardised to unit variance.

**eTable 5.** Dietary quality and methylation-based markers of ageing*

| Dietary quality index | PCGrimAge | PCPhenoAge | ZhangAge | TelomereAgeRev | DunedinPACE |
| --- | --- | --- | --- | --- | --- |
| Dietary inflammatory index (per 1.8 points) | 0.015 (-0.001,0.030), p=0.067 | 0.009 (-0.017,0.034), p=0.505 | -0.013 (-0.046,0.020), p=0.442 | -0.006 (-0.036,0.024), p=0.697 | 0.076 (0.043,0.109), p<0.001 |
| Mediterranean diet score (per 1.8 points) | -0.010 (-0.023,0.004), p=0.159 | 0.006 (-0.016,0.028), p=0.607 | 0.015 (-0.014,0.044), p=0.306 | -0.002 (-0.029,0.024), p=0.879 | -0.032 (-0.061,-0.003), p=0.033 |
| Alternative healthy eating index (per 11 points) | -0.021 (-0.033,-0.009), p<0.001 | -0.015 (-0.035,0.004), p=0.121 | -0.006 (-0.031,0.019), p=0.632 | -0.011 (-0.034,0.012), p=0.358 | -0.058 (-0.084,-0.033), p<0.001 |

* Estimate (95% confidence interval), p value.

Models were fitted independently and were adjusted for age, sex, country of birth, SEIFA decile, blood sample type, case-control study, assay slide, smoking, smoking pack years, education, and self-reported physical activity score. Dietary quality scores are rescaled to units of one standard deviation. Methylation-based ageing markers are standardised to unit variance.

**eTable 6.** Dietary quality and methylation-based markers of ageing*

| term | PCGrimAge | PCPhenoAge | ZhangAge | TelomereAgeRev | DunedinPACE |
| --- | --- | --- | --- | --- | --- |
| Energy (per 3200 kj/day) | -0.016 (-0.027,-0.004), p=0.008 | -0.005 (-0.024,0.014), p=0.587 | -0.015 (-0.039,0.010), p=0.238 | -0.021 (-0.044,0.001), p=0.064 | -0.009 (-0.033,0.016), p=0.489 |
| Coffee (per cup/day) | 0.001 (-0.006,0.008), p=0.791 | -0.007 (-0.018,0.004), p=0.225 | -0.012 (-0.026,0.003), p=0.106 | -0.008 (-0.022,0.005), p=0.217 | -0.003 (-0.018,0.012), p=0.692 |
| Tea (per cup/day) | -0.000 (-0.007,0.006), p=0.953 | 0.006 (-0.005,0.016), p=0.295 | 0.005 (-0.009,0.019), p=0.485 | 0.009 (-0.004,0.022), p=0.168 | -0.008 (-0.022,0.006), p=0.277 |

* Estimate (95% confidence interval), p value.

Models were fitted independently and were adjusted for age, sex, country of birth, SEIFA decile, blood sample type, case-control study, assay slide, smoking, smoking pack years, education, and self-reported physical activity score. Methylation-based ageing markers are standardised to unit variance.

**eFigure 1.** Causal directed acyclic graph for cross-sectional analysis


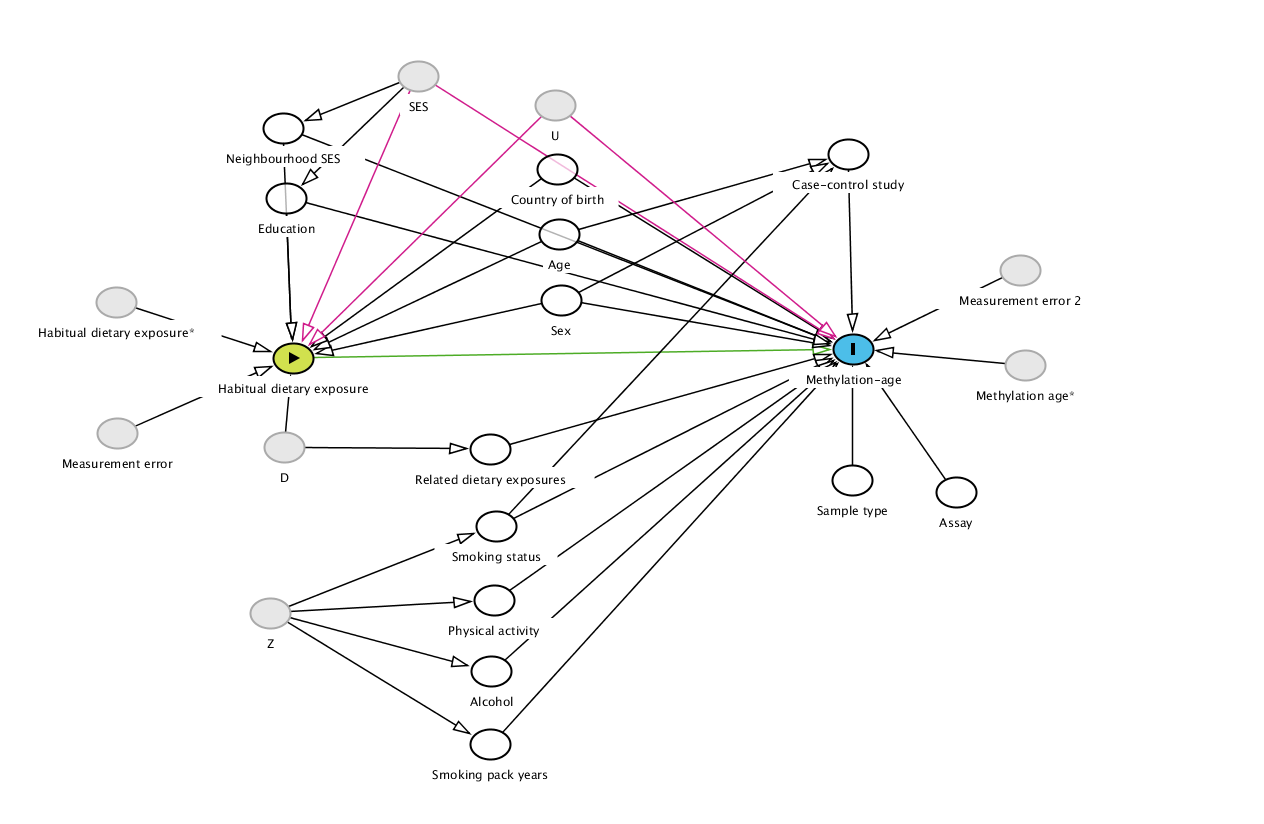


The green node indicates the exposure variable (*Habitual dietary intake*), and the blue node (*Methylation-age*) is the outcome variable. Pale grey nodes indicate unobserved variables; white nodes indicate a variable which has been conditioned on (by regression adjustment or restriction). Paths in red are biasing paths. Arrows indicate the direction of causal effect between two nodes. D represents unmeasured causes of dietary preferences. Z represents unmeasured causes of health behaviours (e.g., personality, genetics). U represents potential unmeasured common causes of diet and methylation-based markers of ageing (e.g., geographical location). Socioeconomic status (SES) is measured by two proxy variables: education and neighbourhood social advantage. Red paths from SES to dietary exposure and methylation age indicates residual confounding resulting from information in SES not captured by measured proxies.

SES = socioeconomic status.

**eFigure 2**. Flow diagram for selection into analysis sample

Full MCCS cohort
(n = 41,513)

Methylation data available
(n = 6,148)

*Providing 6790 unique measurements

Breast cancer study excluded (n = 5,353)

Energy intake within bounds (n = 5,310)

**eFigure 3.** Macronutrients and sub-components and methylation-based markers of ageing (original units)


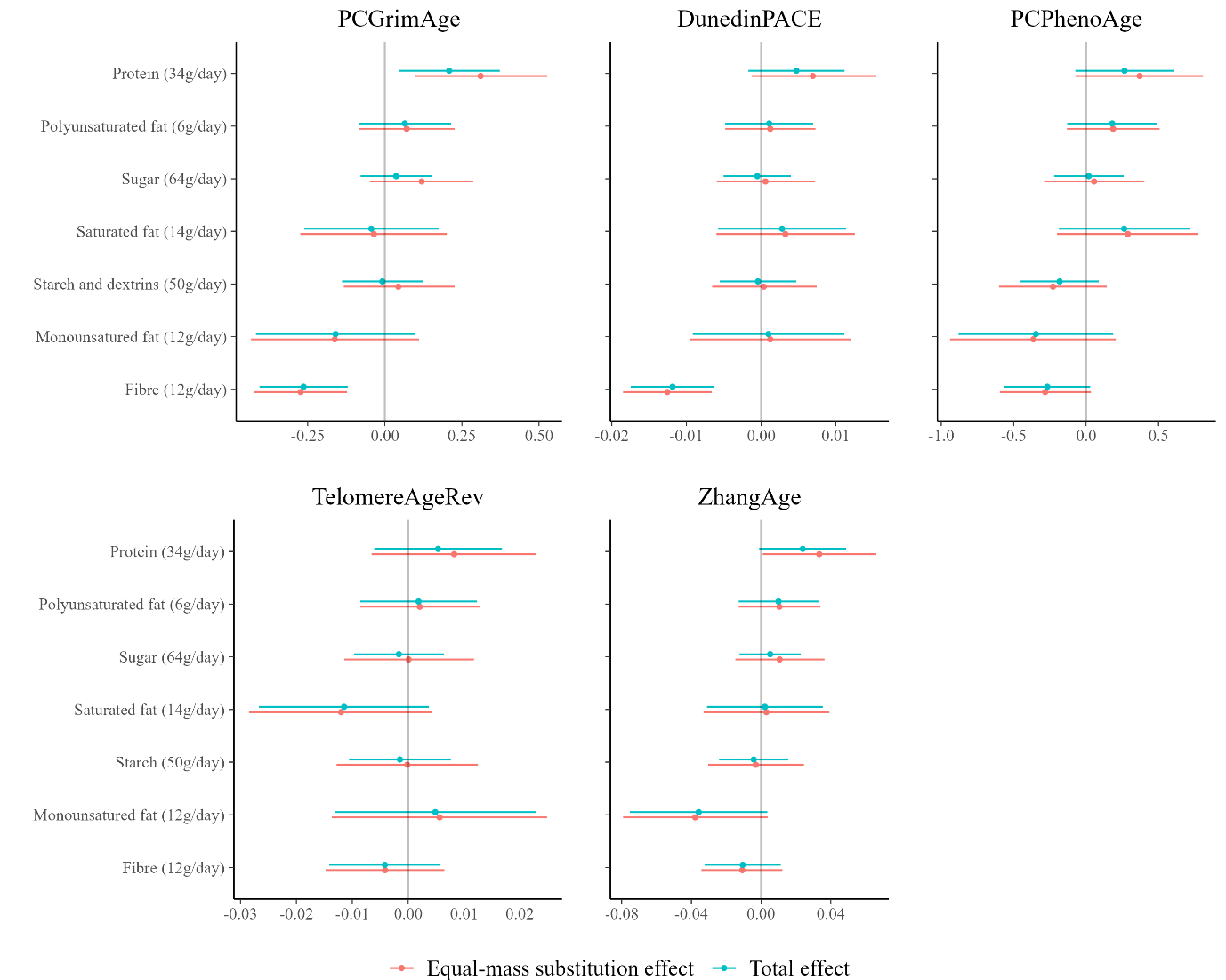


Models included all nutrient exposures and were adjusted for age, sex, country of birth, SEIFA decile, blood sample type, case-control study, assay slide, smoking, smoking pack years, education, and self-reported physical activity score. The equal-mass substitution effect represents the effect of adding a given dietary component to the diet while removing a mass-equivalent amount of the remaining components. The total effect represents the effect of adding a given amount of the dietary component to the diet while holding all other components constant. The X axis is inconsistently scaled across panels for ease of reading.

**eFigure 4a.** Food groups and methylation-based markers of ageing (original units)


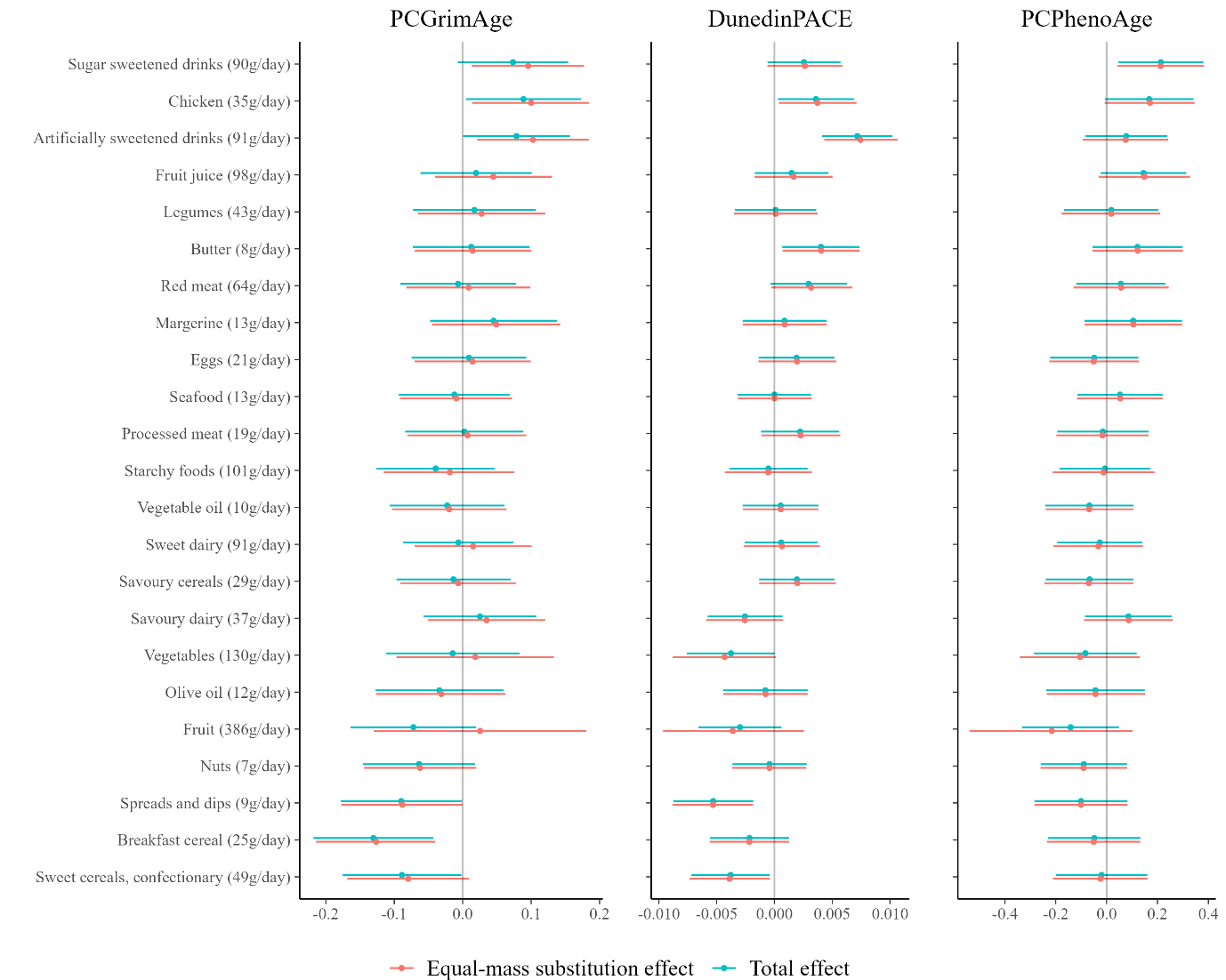


Models included all nutrient exposures and were adjusted for age, sex, country of birth, SEIFA decile, blood sample type, case-control study, assay slide, smoking, smoking pack years, education, and self-reported physical activity score. The average relative causal effect represents the effect of adding a given dietary component to the diet while removing a mass-equivalent amount of the remaining components. The total causal effect represents the effect of adding a given amount of the dietary component to the diet while holding all other components constant. The X axis is inconsistently scaled across panels for ease of reading.

**eFigure 4b.** Food groups and methylation-based markers of ageing (original units)

**
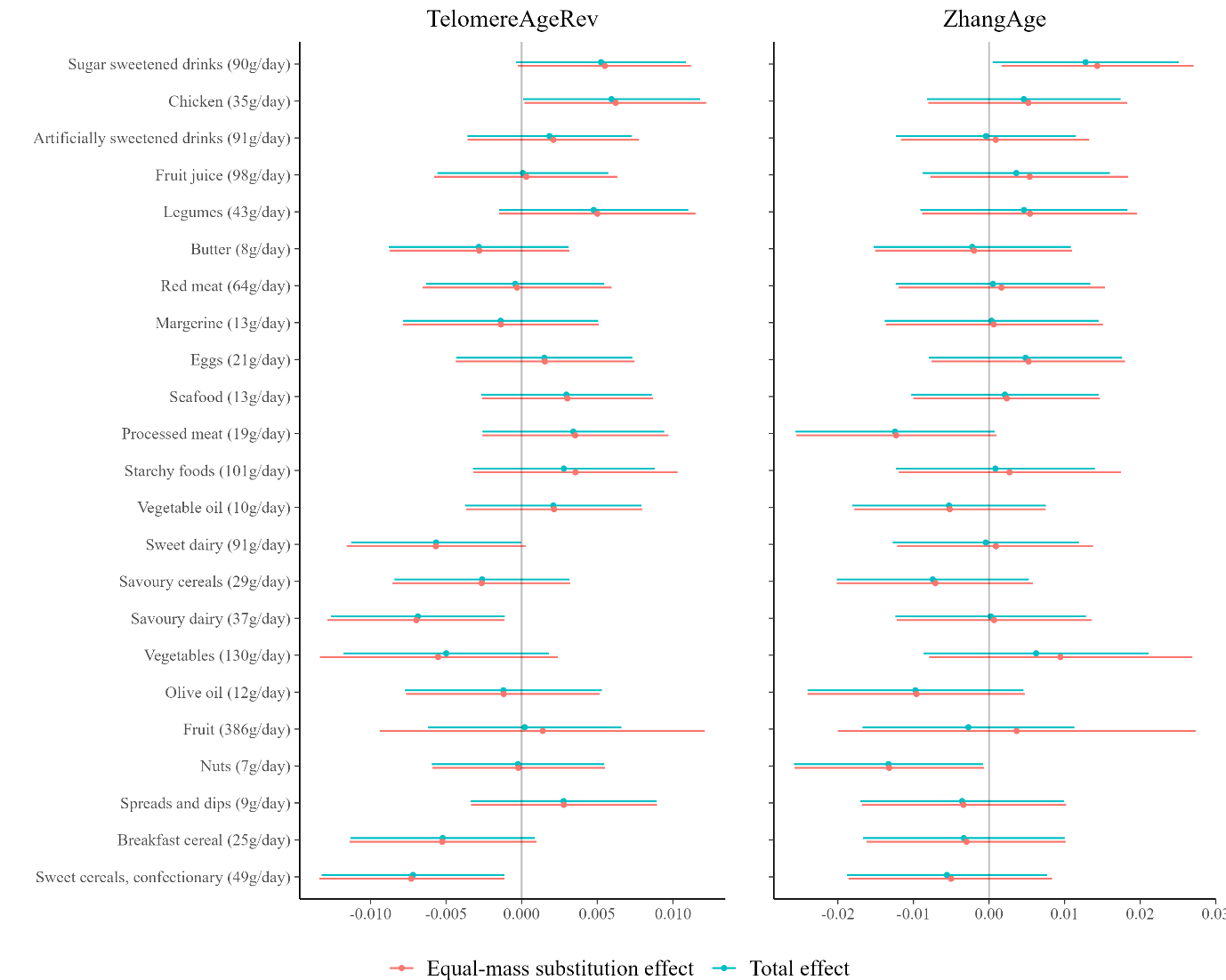
**

**eFigure 5.** Dietary quality and methylation-based markers of ageing (original units)


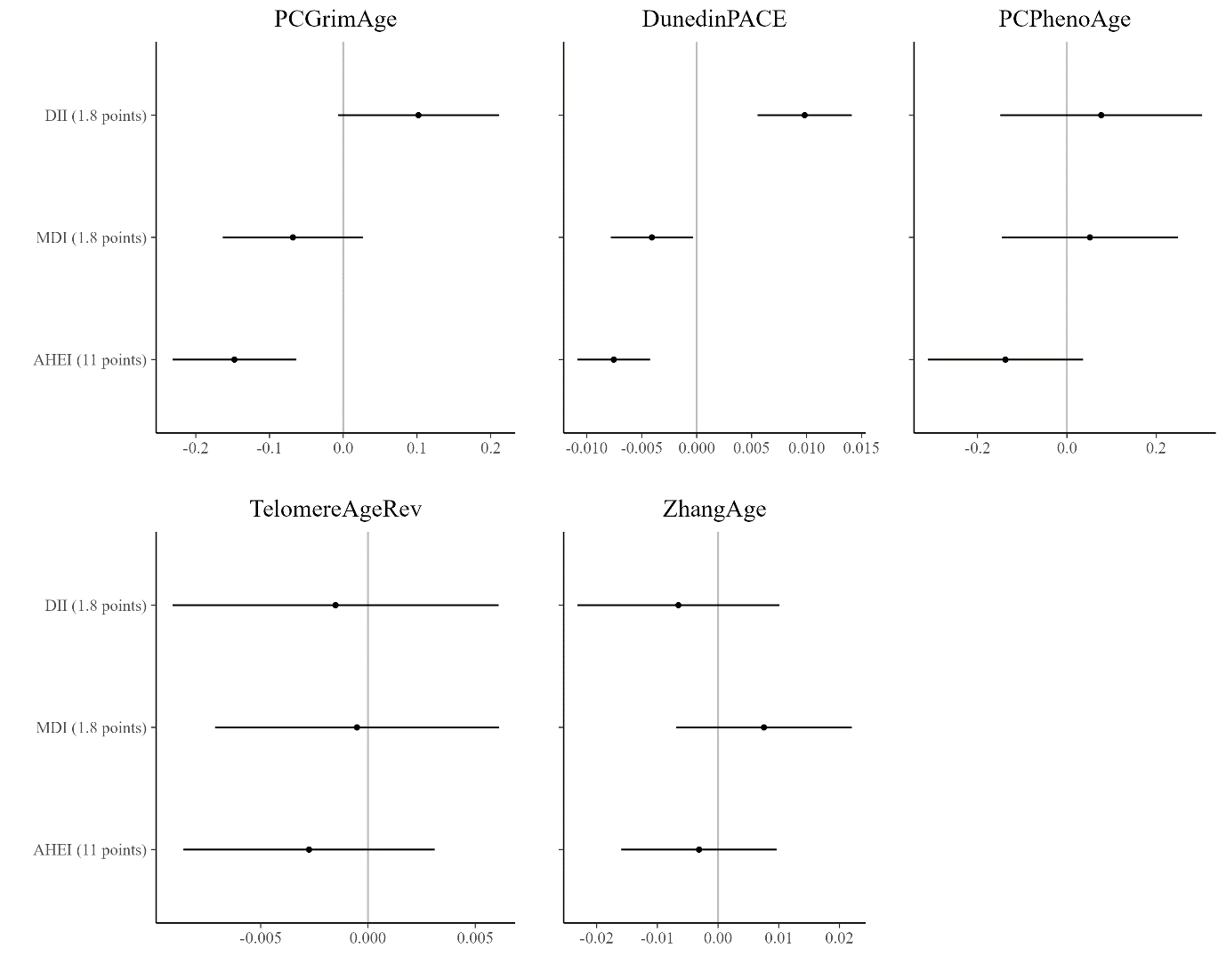


Models were fitted individually for each dietary quality score and were adjusted for age, sex, country of birth, SEIFA decile, blood sample type, case-control study, assay slide, smoking, smoking pack years, education, self-reported physical activity score, and total energy intake.

**eFigure 6.** Coffee, tea, and total energy intake and methylation-based markers of ageing (original units)


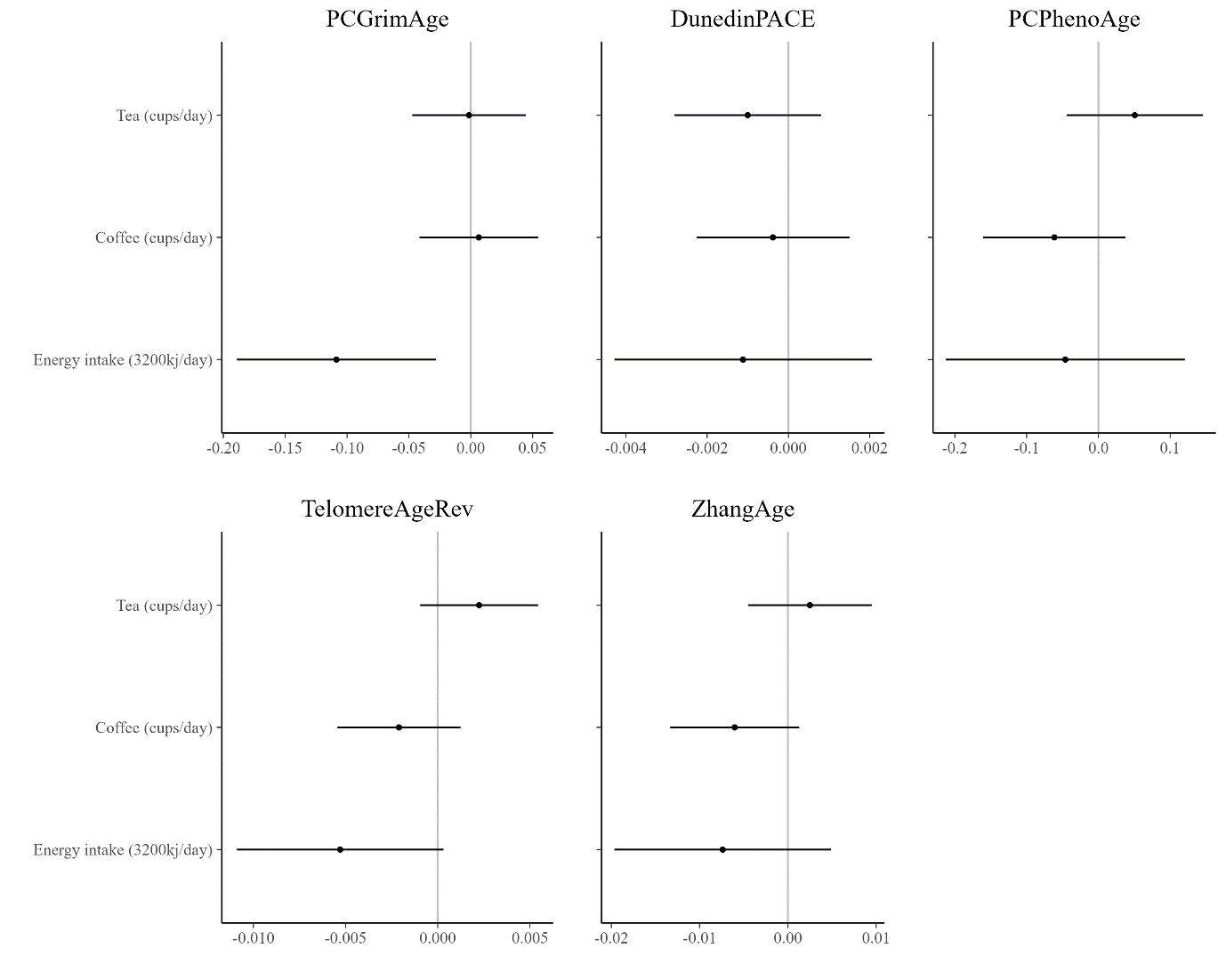


Models were fitted individually for each of tea, coffee, and energy intake, and were adjusted for age, sex, country of birth, SEIFA decile, blood sample type, case-control study, assay slide, smoking, smoking pack years, education, and self-reported physical activity score. Models for coffee and tea are additionally adjusted for dietary quality (AHEI-2010) and total energy intake. Models for total energy intake are additionally adjusted for dietary quality score (AHEI-2010).
